# Current Coverage of the mTOR Pathway by Next-Generation Sequencing Oncology Panels

**DOI:** 10.3390/ijms20030690

**Published:** 2019-02-05

**Authors:** Rita Seeboeck, Victoria Sarne, Johannes Haybaeck

**Affiliations:** 1 Clinical Institute of Pathology, University Hospital St. Poelten, Karl Landsteiner University of Health Sciences, 3100 St. Pölten, Austria; rita.seeboeck@stpoelten.lknoe.at; 2 Department Life Sciences, IMC University of Applied Sciences Krems, 3500 Krems, Austria; victoria.sarne@fh-krems.ac.at; 3 Department of Pathology, Medical Faculty, Otto-von-Guericke University Magdeburg, 39106 Magdeburg, Germany; 4 Department of Pathology, Neuropathology, and Molecular Pathology, Medical University of Innsbruck, 6020 Innsbruck, Austria; 5 Department of Neuropathology, Diagnostic & Research Center for Molecular BioMedicine, Institute of Pathology, Medical University of Graz, 8036 Graz, Austria; 6 German Center for Neurodegenerative Diseases (DZNE), 39120 Magdeburg, Germany

**Keywords:** mTOR, NGS, illumina, IonTorrent, eIFs

## Abstract

The mTOR pathway is in the process of establishing itself as a key access-point of novel oncological drugs and targeted therapies. This is also reflected by the growing number of mTOR pathway genes included in commercially available next-generation sequencing (NGS) oncology panels. This review summarizes the portfolio of medium sized diagnostic, as well as research destined NGS panels and their coverage of the mTOR pathway, including 16 DNA-based panels and the current gene list of Foundation One as a major reference entity. In addition, we give an overview of interesting, mTOR-associated somatic mutations that are not yet incorporated. Especially eukaryotic translation initiation factors (eIFs), a group of mTOR downstream proteins, are on the rise as far as diagnostics and drug targeting in precision medicine are concerned. This review aims to raise awareness for the true coverage of NGS panels, which should be valuable in selecting the ideal platform for diagnostics and research.

## 1. Introduction

### 1.1. mTOR Pathway

The mTOR protein, a serine-threonine kinase of the phosphoinositide 3-kinase (PI3K)-related family, is part of two distinct complexes, mTORC1 and mTORC2. It regulates the cells in all catabolic and anabolic processes dependent on nutrients. Major components of the signaling network are summarized in Table 1 and introduced in the following chapters. Being an anchor point of cell growth, mTOR signaling is a critical target of genetic variation in cancer, and when affected it is frequently associated with carcinogenesis and tumor progression. As its term “mechanistic target of rapamycin” implies, mTOR is the target of the rapamycin-FKB12 complex [1]. mTOR is the catalytic subunit of two protein complexes, known as mTORC1 and mTORC2, acquiring different substrate specificities [2]. mTORC1 consists of a total of five components, apart from mTOR, regulatory-associated protein of mTOR (Raptor), mammalian lethal with Sec13 protein eight (mLST8, also referred to as GβL), proline-rich AKT substrate 40 kDa (PRAS40) and DEP-domain-containing mTOR-interacting protein (Deptor) [2].

Raptor is required for the correct subcellular localization of mTOR and facilitates substrate recruitment to mTOR by binding to the TOR signaling (TOS) motif on mTORC1 substrates [1,2,3]. mLST8 has been proposed to associate with the catalytic domain of the complex and to stabilize the kinase loop [4]. Despite these findings, it was also reported that mLST8 is not essential for mTORC1 signaling [5].

The remaining two subunits, PRAS40 and Deptor, have been characterized as negative regulators [6,7]. In this manner, when mTORC1 activity is reduced, the two subunits are recruited to the complex and promote the inhibition of mTORC1. Furthermore, it has been proposed that PRAS40 functions as a regulator of mTORC1 kinase activity by direct inhibition of substrate binding [8]. When mTORC1 is activated, it phosphorylates PRAS40 and Deptor, thereby reducing the physical interaction with mTORC1 and further activating the complex [7,8].

The second complex, mTORC2, shares some of the same subunits: mTOR, mLST8, and Deptor. Additionally, it consists of the rapamycin-insensitive companion of mTOR (Rictor), mammalian stress-activated protein kinase interacting protein (mSIN1), and protein observed with Rictor-1 (Protor-1). Deptor, again, has been shown to negatively regulate the activity of the complex [7]. In contrast, mLST8 seems to play a crucial role in maintaining mTORC2 function [5]. Rictor and mSIN1 have been reported to stabilize each other, thereby providing the structural foundation of mTORC2 [9,10]. mSIN1 additionally contains a phosphoinositide-binding PH domain that is critical for the insulin-dependent regulation of mTORC2 activity [1]. Rictor has also been shown to interact with Protor-1, but the physiological function of this interaction is not yet clear [11,12].

When considering the upstream signaling of these two complexes, it is important to mention, even though not relevant in physiological conditions, that mTORC1 is considered to be rapamycin-sensitive, whereas TOCR2 is not [13]. When rapamycin enters the cell, it binds to FK506-binding protein 12 kDa (FKBP12) and interacts with the FKBP12 binding domain (FBD) of mTOR. This interaction inhibits the function of mTORC1. On the contrary, rapamycin-FKBP12 cannot acutely inhibit mTORC2 [13]. However, it has been shown that, in some cases, chronic rapamycin treatment can inhibit mTORC2 activity after all [14]. Furthermore, it has been reported that rapamycin does not inhibit all functions of mTORC1 [15].

mTORC1 can be regulated by a variety of signals, such as growth factors (GFs), energy status, oxygen, DNA damage, and amino acids [16]. Multiple different GF pathways converge on one of the most important factors regulating mTORC1, the tuberous sclerosis complex (TSC). TSC is a heterotrimer that consists of TSC1, TSC2, and TBC1D7 [17]. This complex functions as a GTPase activation protein (GAP) for the Ras homolog enriched in brain (Rheb), converting it to its inactive, GDP-bound state. Active, GTP-bound Rheb directly interacts with mTORC1 and stimulates its activity. Hence, TSC1/2 negatively regulates mTORC1 [6,16,18]. GF pathways regulating mTORC1 include the insulin/insulin-like growth factor-1 (IGF-1) pathway, receptor-tyrosine kinase-dependent Ras signaling pathway, as well as Wnt and TNFα signaling. IGF-1 causes AKT-dependent phosphorylation of TSC2 [1,19]. Ras signaling also activates mTORC1 via TSC2 phosphorylation, which is achieved through the MAP kinase ERK and its effector p90RSK [1,16,20]. Additionally, AKT activation can activate mTORC1 in a TSC1/2-independent manner, by the promotion of the dissociation of PRAS40 from mTORC1 [6,8,21]. Wnt and TNFα, on the other hand, exert their influence on mTORC1 via the inhibition of TSC1 [22,23].

Intracellular and extracellular stress signals can also regulate mTORC1. In this manner, a reduction in cellular energy activates AMPK, which inhibits mTORC1 through the phosphorylation of Raptor and the activation of TSC2 [1,22]. Hypoxia also activates AMPK, but affects mTORC1 additionally through the induction of REDD1, which activates TSC [24]. DNA damage inhibits the mTORC1 complex via the induction of p53 target genes which, in turn, increase TSC activity [25]. Amino acid sensing by mTORC1 is mediated by Rags, which are heterodimers consisting of RagA or RagB with RagC or RagD [26]. These dimers are tethered to the lysosomal membrane [27,28]. Upon amino acid stimulation, Rags are activated and bind Raptor, leading to the recruitment of mTORC1 to the lysosomal membrane where Rheb is located as well [28]. mTORC1 signaling only takes place when both Rheb and Rags are activated [1].

**Figure 1:**
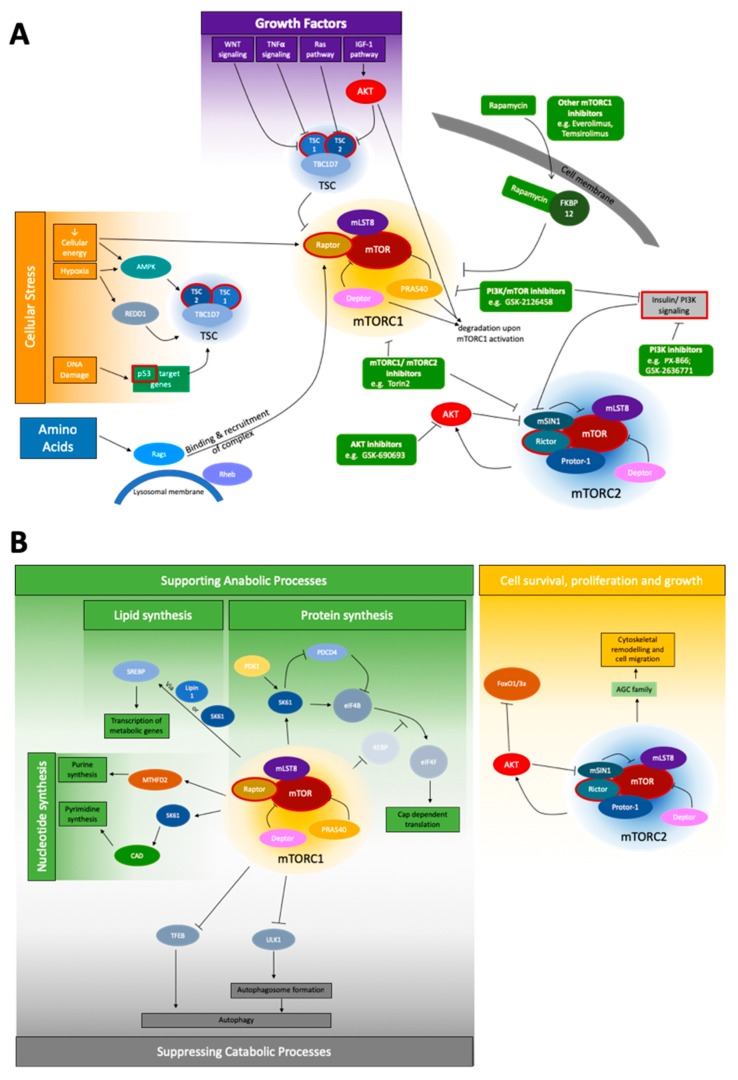
Schematic depiction of the mTOR signaling network. (**A**) The most important upstream signals of mTOR signaling are cellular stress, growth factors, and amino acids for the mTORC1 complex, and the insulin/PI3K pathway for mTORC2. Genes of special interest for NGS, due to their mutation frequency, are shown with a red border. No prominent pattern of a section specifically affected by these mutations is obvious. To the best of our best knowledge, no companion diagnostic between a specific mutation and treatment is currently applicable. However, a variety of inhibitors (green) affecting the network at different points are known: Inhibitors affecting AKT, mTORC1/mTORC2, PI3K, PI3K/mTOR, and mTORC1 inhibitors like rapamycin and rapalogs [51,52]; and (**B**) the widespread downstream network of mTORC1 and mTORC2 is shown. mTORC1 is involved both in supporting anabolic processes via influencing nucleotide, lipid and protein synthesis, as well as suppressing catabolic processes, mainly autophagy. mTORC2, under the coregulation of AKT, is known to mainly affect cell survival, proliferation and growth, and the specifically the regulation of the cytoskeleton.

Compared to the mTORC1 upstream network, mTORC2 seems to be less complex. The complex primarily functions as an effector of insulin/PI3K signaling [1]. The PH domain of mSIN1 inhibits the catalytic function of mTORC2 in the absence of insulin. Upon binding to PI3K-generated PIP3, this inhibition is relieved [29]. Furthermore, AKT can phosphorylate mSIN1, suggesting the presence of a positive feedback loop in which partial AKT activation promotes mTORC2 activation, which then fully activates AKT [30]. Another regulator of mTORC2 is mTORC1, mediated by a negative feedback loop between mTORC1 and insulin/PI3K signaling [1,31]. The upstream network, as described here, is illustrated in Figure 1A.

The downstream signaling of mTORC1 is as diverse as its upstream paths. It plays a crucial role in the balance between anabolism and catabolism by promoting lipid, protein, and nucleotide production while simultaneously suppressing autophagy [1].

Lipid synthesis is promoted by mTORC1 through the sterol responsive element binding protein (SREBP) transcription factors. These control the expression of metabolic genes [1,32]. Usually SREPB is activated due to low sterol levels. However, mTORC1 can activate SREPB independent of sterol levels in a p70S6 Kinase 1 (S6K1)-dependent manner or via the phosphorylation of another substrate, Lipin1. In the absence of mTORC1, Lipin1 inhibits SERBP [33,34].

mTORC1 mostly promotes protein synthesis via S6K1 phosphorylation and eukaryotic initiation factor 4E (eIF4E) Binding Protein-1 (4EBP-1). By phosphorylating S6K1, mTORC1 enables its subsequent activation by PDK1. The active S6K1 then activates factors in favor of mRNA translation initiation, including eIF4B, which is a positive regulator of the 5’ cap binding eIF4F complex [35]. S6K1 is known to phosphorylate, thereby promoting the degradation of PDCD4, an inhibitor of eIF4B [36]. 4EBP inhibits translation by binding eIF4E, which prevents the assembly of the eIF4F complex. However, mTORC1 phosphorylates 4EBP and thereby causes its dissociation from eIF4E. This allows eIF4E to promote cap-dependent translation [37,38,39]. This tremendous regulatory power of translation initiation links these two molecular processes and requires a consideration of further eIF subunits, when looking at the overall impact of mTOR signaling. Taking all subunits into consideration, this protein family comprises more than 30 members [40].

The synthesis of nucleotides is also promoted by mTORC1 signaling. Thus, mTORC1 induces purine synthesis through the increased expression of MTHFD2, which controls the mitochondrial tetrahydrofolate cycle [41]. In a similar vein, S6K1 phosphorylates carbamoyl-phosphate synthetase (CAD), which catalyzes the initial steps of de-novo pyrimidine synthesis [42]. mTORC1 has also been shown to increase the translation of HIF1α, which leads to the expression of glycolytic enzymes such as PFK [33]. The activation of SREBP by mTORC1 additionally leads to an increase in the pentose phosphate pathway (PPP). These mechanisms lead to a shift in glucose metabolism towards glycolysis, which facilitates growth [1]. All these anabolic processes support cell growth, however, mTORC1 also supports growth by the suppression of catabolic processes, the most notable process being autophagy [1]. An important transcription factor that drives the expression of genes for autophagy and lysosomal biogenesis is the transcription factor EB (TFEB). This transcription factor can be phosphorylated by mTORC1, which subsequently inhibits its nuclear translocation [43]. Furthermore, another important step in autophagy can be inhibited by mTORC1, which is the autophagosome formation. ULK1, a kinase that normally forms a complex with a number of other components, drives the autophagosome formation. However, under nutrient-rich conditions, mTORC1 phosphorylates ULK1 and thereby disrupts the interaction between ULK1 and AMPK, which is an activator of autophagy [44].

Similar to the upstream processes, the downstream processes of mTORC2 are considered to be less complex. Most probably, mTORC2 plays a key role in the phosphorylation of AKT [1]. AKT is a key effector protein of the insulin/PI3K signaling pathway and can be activated by mTORC2 [45]. When active, AKT promotes cell proliferation, survival and growth through the inhibition of various substrates, including but not limited to the FoxO1/3a TFs; GSK3ß, a metabolic regulator; and TSC2 [45]. Nevertheless, studies have shown that mTORC2 is not crucial to the phosphorylation of all substrates of Akt, for example, TSC can be phosphorylated by Akt without mTORC2 [5]. However, it has been reported to be essential for the phosphorylation of other substrates, such as FoxO1/3a [10]. mTORC2 phosphorylates several members of the AGC (PKA/PKG/PKC) family and thereby controls proliferation and survival [46,47]. One major cellular process influenced by mTORC2 is the actin cytoskeleton. Several PKC family members have been reported to be phosphorylated by mTORC2, all of which are involved in the regulation of cytoskeletal remodeling and cell migration [46,47,48,49]. Furthermore, mTORC2 can also activate SGK1, which is an AGC-kinase that regulates ion transport and cell survival [50].

The downstream processes of mTOR signaling are shown in Figure 1B. All proteins involved in mTOR signaling are summarized in Table 1.

### 1.2. mTOR Signaling in Cancer

Considering how involved the mTOR signaling pathway is, it comes as no surprise that it also plays a crucial role in human disease, particularly cancer. The complex most commonly associated with cell proliferation and cancer progression when deregulated is the mTORC1 complex [53,54]. A number of signaling components both upstream and downstream of mTOR are frequently deregulated or altered in human cancer [53]. Through alterations in one or multiple of these elements, mTOR signaling is activated in many cancer types, suggesting mTOR as a potent target for cancer therapy. Due to this fact, mTOR pathway inhibitors have been of prime interest in recent years. These inhibitors include rapamycin and its analogs (rapalogs) and, more recently, mTOR kinase domain inhibitors [55]. Despite showing promise, rapalog monotherapy has been proven mostly insufficient in causing tumor regression, with notable exceptions of tumors showing mutations in mTOR itself, LOF mutations in TSC1 or TSC2 [55,56,57,58,59]. Broadrange reports correlating mTOR pathway mutations to drug response are yet missing, but there are studies towards that aim that are very promising. Specifically, a study identified 33 *MTOR* mutations that lead to pathway hyperactivity in cancer [58]. A heightened rapamycin sensitivity in cells harboring these hyperactivating mTOR mutations suggests that they convey mTOR pathway dependency. These results are supported by the report of an extraordinary responder with two activating mTOR mutations in urothelial carcinoma and an exceptional response to rapalog treatment in combination with a TKI [58,59].

Furthermore, patients with the genetic disorder tuberous sclerosis complex (TSC) (mutations in the *TSC1* or *TSC2* gene), commonly develop tumors like astrocytomas or angiomyolipomas as well as the related lung disorder Lymphangioleiomyomatosis (LAM). Treatment with rapalogs has been shown to improve clinical outcomes and cause tumor regression in TSC patients with astrocytomas or sporadic LAM, again suggesting a dependence on mTOR signaling for tumor growth [60,61,62]. A phase II clinical trial found a 50% response rate in TSC patients with angiomyolipomas or sporadic LAM [63]. Furthermore, heightened treatment sensitivity was associated with TSC1 or TSC2 LOF mutations, as reported in bladder and thyroid cancer [56,57]. Other responders have been reported in one pancreatic cancer with loss of suppression of mTOR signaling and three patients with perivascular epithelioid cell tumors with the loss of TSC2 [64,65]. However, in the thyroid cancer extraordinary responder case study, the tumor gained resistance to rapalog treatment as it acquired a mutation in mTOR, which prevented the binding of the rapalog, as well as a nonsense mutation in TSC2 [57]. Further literature regarding rapamycin and rapalogs as monotherapy includes References [66] and [67]. These specific cases show the importance of rapamycin and rapalogs, as well as the development of reliable biomarkers, for precision medicine. Apart from these cases, it has been shown that, while not very potent on its own, mTORC1 inhibition might be necessary to achieve a proper response to drugs that target the primary oncogenic pathway in the given cancer. On top of that, sustained mTORC1 activation is proposed to be a major mechanism of resistance to targeted therapies [55,56,57,58,59,68].

Furthermore, mTORC1 is, as mentioned above, not only involved in stimulating growth but also in regulating autophagy. Autophagy has been described as double-edged sword in the modulation of cancer, since both inhibition and induction of autophagy have been shown to be both pro and anti-tumorigenic [54,55,56,57,58,59,68,69]. Even though a better understanding of the individual factors contributing to the effect autophagy has on cancer is needed, mTORC1 and its associated regulators of autophagy, ULK1 and AMPK, represent attractive targets for cancer therapy [54].

### 1.3. Next-Generation-Sequencing

DNA sequence analysis has come a long way since the establishment of the Sanger chain termination method in 1977 [70]. From then on, scientists have developed reliable and reproducible ways of DNA sequencing, steadily decreasing the costs and increasing output. Output, which was formerly one read of one gene at a time, is now more adequately given in gigabases per run, reflecting the parallel analysis of multiple genes with read depths (i.e., the number of reads covering a genetic locus) of 20 up to 1000 or more, depending on the application [71]. Next Generation Sequencing (NGS) is the most common name of the second-generation, deep-sequencing techniques. All platforms are following a three-step procedure: (1) Library-preparation, (2) Cluster/Bridge Amplification, and (3) sequencing, i.e., strands of fragmented DNA are amplified and immobilized on a surface or bead, then nucleotide bases are added sequentially using DNA polymerase; excess reagent is washed out to enable correct imaging according to the base incorporated; this process repeats for each base. The actual sequence analysis is for, e.g., Illumina based on fluorescent signaling, while Ion Torrent technology relies on pH changes detected by semiconductors [72,73,74].

## 2. Summary and Comparison of Oncological NGS Panels and Their Coverage of the mTOR Pathway

In the following, we summarize commercially available NGS gene panels that cover a number of genes reasonable for research and clinical applications, i.e., covering a medium number of gene loci, excluding large scale screening panels. We included the gold standard genetic analysis panel Foundation One as a reference. In a next step, we look at the coverage of the mTOR pathway by the various panels. Therefore, we submerged a 78-item list of mTOR signaling-relevant genes. This list is based on the publicly available “mTOR Pathway—Gene List”, generated with the help of David Sabatini, a leading expert in the field [75]. We extended the list by a number of genes, among them the complete eIF3 and eIF4 protein families, representing a more general field of mTOR impact (Supplementary Table S1).

### Oncological NGS Gene Panels

The growing capacity of NGS devices, with an increasing number of genes and read depth has initiated a trend towards whole exome and whole genome sequencing. These techniques will be state of the art in the near future. Today, bioinformatic and data storage issues also limit the application of global analyses and make panels comprising 10–150 genes to the standards in the field. Most of these panels run on Illumina MiniSeq, MiSeq, and iSeq devices or on the Ion Torrent S5 Series devices by Applied Biosystems [76]. Supplementary Table S1 shows a collection of 16 different gene panels for oncological application and the full gene lists together with the gene list of Foundation One. The gene panels and number of genes are summarized in Table 2. As already mentioned, we consider only ready-made gene panles with a low-medium number of genes analyzed. All these panels are based on DNA only, as DNA material is sufficient to detect genetic mutations. RNA sequencing would add a surplus on information on e.g. gene fusions or gene expression, but RNA is more difficult to isolate from especially FFPE tissue in an adequate quality [77] and besides that hardly any gene panels covering RNA targets are available to date. One of the available gene panels with DNA and RNA pools is the Ion AmpliSeq/AmpliSeq for Illumina Focus Panel, which is also considered in this review. This Focus Panel targets 40 DNA sites and additional 23 RNA sites, among the latter is also the mTOR-relevant AKT3 [78].

The mTOR-relevant genes covered by the oncological NGS panels primarily consist of mTOR upstream AKT and PIK3CA. To elucidate the relevance for the signaling pathway, we collected data from the catalog of somatic mutations in cancer (COSMIC; cancer.sanger.ac.uk/cosmic) on mutational frequency and associated drug sensitivity/resistances (Table 3).

Of the genes analyzed here, TP53 was identified by this catalog as the by far most commonly mutated gene in cancer (25.2%), followed by PIK3CA (9.7%) and PTEN (5%). According to the catalog, none of the genes harbor drug-associated resistance mutations, but indeed, mutations were associated with altered sensitivity. In this manner, mutations of NF1 alter the sensitivity to the drug Nutlin-3a, which is targeting MDM2. Mutations in PIK3CA are associated with altered sensitivity to Pictilisib and GSK690693, targeting PI3K and AKT1/2/3, respectively. Mutations in PIK3R1 are associated with altered sensitivity to Dacinostat, targeting HDAC1. Mutations in PTEN are associated with altered sensitivity to GSK690693. Mutations in TP53 are associated with altered sensitivity to the following seven drugs: 5-Fluorouracil (5-FU, antimetabolite), Rucaparib (targeting PARP1/2), CX-5461 (acts on RNA polymerase 1), (5Z)-7-Oxozeaenol (targeting TAK1), Bleomycin (acts by induction of dsDNA breaks and DNA damage repair), Dabrafenib (targeting BRAF), and Nutlin-3a [79].

The most frequently mutated genes, together with affected tissues and actual occurring mutations, are listed in Table 4.

## 3. Discussion and Conclusions

We have shown that mTOR-associated genes generally show low mutational frequencies in cancer. Only TP53, with 25.2% is a frequent target of mutations, and is known to interact with numerous signaling cascades besides the mTOR pathway. PIK3CA with 9.7% and PTEN with 5% mutational frequency are especially interesting, as they are also associated with drug sensitivities. In fact, a ranking of genes according to their associated drug sensitivity also shows a better representation of the mTOR pathway than with actual number of mutated samples. When looking at all described genes, it becomes evident that very little awareness is drawn to mTOR downstream, e.g., eIFs, with low mutational frequencies throughout and no reported drug sensitivity alterations. This results in a ambivalent situation; on the one hand the high importance of mTOR pathway and translational control for carcinogenesis and growth control of tumor cells, is emphasized by a growing number of research as well as clinical reports, on the other hand, NGS and following the information of tumor mutational burden, generated by NGS analyses show only limited applicability in terms of mTOR pathway associated readouts. For the here featured pathway, it will be critical to employ RNA-sequencing and nanopore sequencing techniques, which will allow for an evaluation of gene expression next to mutational status, thereby multiplying the information on mTOR signaling in cancer. By those means well described predictive as well as prognostic tumor markers can be evaluated by their expression levels. This is changing the view on our gene panel, which holds numerous marker genes that are known to have great impact on disease progression and prognosis, even though they are poorly covered by NGS and are rarely mutated. Important examples of these markers are the eIF subunits [104,105,106].

## Figures and Tables

**Table 1:** mTOR pathway-associated proteins, divided into mTOR complex components and upstream/downstream modules. Where applicable, protein activity is denoted as anabolic or catabolic.

Abbreviation	Full Name	Function	↑↓
mTORC 1		Stimulating/Inhibiting Signal	
mTOR	mechanistic target of rapamycin	Serine-threonine kinase	-
Raptor	regulatory-associated protein of mTOR	Localization of mTOR, substrate recruitment to mTOR [1,3,4]	↑
mLST8	mammalian lethal with Sec13 protein 8	Stabilizing kinase loop [5]; not essential to TORC1 function [6]	-
PRAS40	proline rich AKT substrate 40 kDa	Inhibitory [7]; inhibits substrate binding, phosphorylated by active mTORC1 [9]	↓
Deptor	DEP-domain-containing mTOR-interacting protein	Inhibitory [8], phosphorylated by active mTORC1	↓
mTORC 2		Stimulating/Inhibiting Signal	
mTOR	mechanistic target of rapamycin	serine-threonine kinase of the phosphoinositide 3-kinase (PI3K)-related family	
mLST8	mammalian lethal with Sec13 protein 8	Essential for stability and function of mTORC2 [6]	↑
Deptor	DEP-domain-containing mTOR-interacting protein	Inhibitory [8]	↓
Rictor	rapamycin-insensitive companion of mTOR	Stabilization [10,11]; shown to interact with Protor-1 [12,13]	↑
mSIN1	mammalian stress-activated protein kinase interacting protein	Stabilization [10,11], phosphoinositide-binding PH domain: critical for insulin dependent mTORC2 function, inhibits mTORC2 function in absence of insulin [1]	↑/↓
Protor-1	protein observed with Rictor-1	shown to interact with Rictor [12,13]	
mTORC 1 Upstream		Stimulating/Inhibiting Signal	
-	rapamycin	Enters cell and binds FKBP12 [2]; when bound inhibits mTORC 1, but not all functions [15]	↓
FKBP12	FK506-binding protein 12 kDa	Is bound by rapamycin, interacts with FBD on mTOR [2]; when bound inhibits mTORC 1, but not all functions [15]; cannot acutely inhibit mTORC2 [2]	-
TSC	tuberous sclerosis complex	Consists of TC1, TC2, TBC1D7, negatively regulates mTORC1 via inactivation of Rheb [17], phosphorylated by AKT (mTORC2 independent) [6]	↓
Rheb	Ras homolog enriched in brain	Stimulates mTOCR1 activity when active [7,16,18]	↑
IGF-1 pathway	insulin/insulin like growth factor 1 pathway	Causes AKT dependent phosphorylation of TSC2 [1,19]	↑
Ras pathway	Rat Sarcoma Pathway	Causes TSC2 phosphorylation via ERK and p90rsk [1,16,20]	↑
AKT	AKT serine/threonine kinase	Phosphorylates TSC2 [1,19]; key effector protein of the insulin/PI3K signaling pathway, and can be activated by mTORC2 [45]; promotes dissociation of PRAS40 from mTORC1. [7,9,21]	↑
-	Wnt	Inhibits TSC1 [22]	↑
TNFα	tumor necrosis factor α	Inhibits TSC1 [23]	↑
AMPK	5’-AMP-activated protein kinase	Inhibits mTORC1 (in response to reduced cellular energy or hypoxia) by phosphorylating Raptor and activation of TSC2 [1,22,24]; activator of autophagy, activates ULK1 [44]	↓
REDD1	regulated in development and DNA damage responses 1	Activates TSC in response to hypoxia [24]	↓
-	p53 target genes	Increase TSC activity upon DNA damage [25]	↓
mTORC 2 Upstream		Stimulating/Inhibiting Signal	
	Rapamycin	Enters cell and binds FKBP12 [2];	↓
FKBP12	FKBP prolyl isomerase	Is bound by rapamycin, interacts with FBD on mTOR cannot acutely inhibit mTORC2 [2]; chronic treatment can inhibit mTORC2 [14]	-
PIP3	Phosphatidylinositol (3,4,5)-trisphosphate	PI3K generated PIP3 binds to PH domain o mSIN1 and relieves inhibition of mTORC2 [29]	↑
AKT	AKT serine/threonine kinase	Phosphorylates mSIN1, positive feedback loop [30]	↑
mTORC1	mammalian target of rapamycin complex 1	Negative feedback loop between mTORC1 and insulin/PI3K signaling [1,31]	↓
mTORC1 downstream		Stimulating/Inhibiting Signal	
SREBP	sterol responsive element binding protein	Activated by low sterol levels, in control of expression of metabolic genes, can be activated by mTORC1 independently via S6K1 or Lipin1 [1,32]; expression by mTORC1 increases PPP [1]	↑
S6K1	p70S6 Kinase 1	Can activate SREBP [33,34], when phosphorylated by mTORC1 can be activated by PDK1, promotes mRNA translation intitation [35]; promotes degradation of PDCD4 [36]; phosphorylates CAD (catalyzes first steps in de-novo pyrimidine synthesis) [42]	↑
-	Lipin1	Inhibits SREBP in absence of mTORC1, activates when mTORC1 is present [33,34]	↑
4EBP	eukaryotic initiation factor 4E binding protein	Inhibits translation by binding eIF4E → prevents assembly of eIF4F complex; when phosphorylated by mTORC1 → dissociates from eIF4e → allows assembly [37,38,39]	↑
eIF4B	eukaryotic translation initiation factor 4B	Positive regulator of the 5’-cap binding eIF4F complex, activated by S6K1 [35], inhibitor: PDCD4 [36]	↑
eIF4F complex	eukaryotic translation initiation factor 4F	Positively regulated by eIF4B, 5’-cap binding complex,	↑
MTHFD2	methylenetetrahydrofolate dehydrogenase	Controls the mitochondrial tetrahydrofolate cycle, expression increased bymTORC1 induction of purine synthesis [41]	↑
HIF1α	hypoxia inducible factor 1	Translation increased by mTORC1 → expression of glycolytic enzymes [33]	↑
TFEB	Transcription factor EB	expression of genes for autophagy and lysosomal biogenesis, when phosphorylated by mTORC1 →cannot translocate to nucleus [43]	↓
ULK1	unc-51 like autophagy activating kinase 1	Drives autophagosome formation, when phosphorylated by mTORC1 → no interaction with AMPK → no activation [44]	↓
AMPK	AMP-activated protein kinase	Inhibits mTORC1 (in response to reduced cellular energy or hypoxia) by phosphorylating Raptor and activation of TSC2 [1,22]; activator of autophagy, activates ULK1 [44]	↓
mTORC2 downstream		Stimulating/Inhibiting Signal	
AKT	AKT serine/threonine kinase	Phosphorylated by mTORC2 [1]; phosphorylates TSC2; key effector protein of the insulin/PI3K signaling pathway [45]; activation by mTORC2 not crucial for the phosphorylation of all, but some of its substrates [6,11]	-
FoxO1/3a	Forkhead box protein O1	TFs, phosphorylated by AKT (mTORC2 dependent) [11]	-
GSK3ß	Glycogen synthase kinase 3β	Metabolic regulator, phosphorylated by AKT [6]	-
-	AGC (PKA/PKB/PKC) Family	Several members phosphorylated by mTORC2 for regulation of proliferation, survival and cytoskeleton [1,46,47,48,49]	-

**Table 2:** Oncologically relevant, predesigned NGS gene panels, with number of genes covered and the names of covered genes relevant to mTOR signaling.

Panel Name	Number of Genes Covered	mTOR Relevant Genes Covered
Foundation One	305	AKT1/2/3; CCND1; GSK3B; MDM2; MTOR; NF1; PDK1; PIK3C2; PIK3CA/B; PIK3R1; PTEN; RICTOR, RPTOR; SGK1; TNFAIP3; TP53; TSC1/2; VHL
Agilent ClearSeq Comprehensive Cancer Panel	150	AKT1/2/3; NF1; MTOR; PIK3R1; PIK3CA; PTEN; TP53; VHL
Qiagen Human Cancer Predisposition GeneRead DNAseq Targeted Panel V2	143	AKT1; NF1; PIK3CA; PTEN; TP53; TSC1/2; VHL
Integrated DNA Technologies (IDT) xGen Pan-Cancer Panel	127	AKT1; CCND1; EIF4A2; MTOR; NF1; PIK3CA; PIK3CG; PIK3R1; PTEN; TP53; VHL
Archer VariantPlex Solid Tumor	67	AKT1; CCND1; MDM2; PIK3CA; PIK3R1; PTEN; TP53; VHL
Swift Biosciences Accel-Amplicon 56G Oncology Panel v2	56	AKT1; PIK3CA; PTEN; TP53; TSC1; VHL
NEBNExt Direct Cancer HotSpot Panel	50	AKT1; PIK3CA; PTEN; TP53; VHL
AmpliSeq Cancer Hotspot Panel v2	49	AKT1; PIK3CA; PTEN; TP53; VHL
TruSeq Amplicon Cancer Panel	48	AKT1; PIK3CA; PTEN; TP53; VHL
AmpliSeq for Illumina Focus Panel	40	AKT1; CCND1; MTOR; PIK3CA
Archer VariantPlex Comprehensive Thyroid and Lung Kit	31	AKT1; CCND1; MDM2; PIK3CA; PTEN; TP53
TruSight Tumor 26	26	AKT1; PIK3CA; PTEN; TP53
Agilent SureMASTR Tumor Hotspot	25	AKT; PIK3CA; PTEN
Qiagen Human Clinically Relevant Tumor GeneRead DNAseq Targeted Panel V2	24	AKT1; PIK3CA; PTEN; TP53
Asuragen QuantideX NGS DNA Hotspot 21 Kit	21	AKT1/2; PIK3CA
TruSight Tumor 15	15	AKT1; PIK3CA; TP53
Qiagen Human Tumor Actionable Mutations GeneRead DNAseq Targeted Panel v2	8	-

**Table 3:** COSMIC data for mTOR pathway-associated genes. The mutational frequencies are highlighted in grey, if >1%. If a gene mutation alters the sensitivity to drug treatment, the gene name is written in bold letters.

Gene	Frequency of Mutation in Cancer
4E-BP	<0.1%
AKT1	1.1%
AKT2	0.4%
AKT3	0.5%
CCND1	0.3%
Deptor	0.3%
eIF3a	0.8%
eIF3b	0.4%
eIF3c	<0.1%
eIF3d	0.3%
eIF3e	0.3%
eIF3f	0.2%
eIF3g	0.2%
eIF3h	0.2%
eIF3i	0.2%
eIF3j	0.1%
eIF3k	0.1%
eIF3l	0.3%
eIF3m	0.2%
eIF4a	0.3%
eIF4b	0.3%
eIF4E	0.2%
eIF4g	1.0%
eIF4h	0.2%
FOXO	0.4%
GBL/mLST8	0.2%
GSK3A	0.2%
GSK3B	0.4%
HIF1α	0.5%
LKB1	0.5%
MDM2	0.4%
mSin1 = MAPKAP1	0.2%
MTHFD2	0.1%
mTOR	2.1%
**NF1**	3.8%
PDK1	0.2%
**PIK3CA**	9.7%
PIK3CB	0.7%
PIK3CD	0.7%
PIK3CG	1.6%
**PIK3R1**	1.4%
PIK3R2	0.5%
PIK3R3	0.3%
PIK3R4	0.7%
PIK3R5	0.7%
PIK3R6	0.6%
PIP3	0.4%
PKC alpha	0.4%
PKC beta	1.0%
PKC delta	0.4%
PKC epsilon	0.5%
PKC eta	0.5%
PKC gamma	0.7%
PKC iota	0.4%
PKC theta	0.7%
PKC zeta	0.4%
PRAS40 = AKT1S1	0.2%
Protor = PRR5	0.3%
**PTEN**	5.0%
Raptor	1.0%
REDD1 = DDIT4	0.1%
Rheb	0.1%
Rictor	1.0%
RRAGA	0.1%
RRAGB	0.2%
RRAGC	0.2%
RRAGD	0.2%
S6K	0.2%
SGK	0.4%
SREBP	0.5%
TFEB	0.3%
TNFα	0.3%
**TP53**	25.2%
TSC1	1.2%
TSC2	1.7%
ULK1	0.7%
VHL	4.5%
Wnt	0.3%

**Table 4:** Summary of most frequently mutated mTOR related genes and affected tissues. The mutational frequencies are highlighted in grey, if >1%.

Gene	Frequency of Mutation in Cancer	Most Common Genetic Mutations	Tissue	Reference
AKT1	1.1%	E17K, Q79K, L52R	breast, skin, urinary tract	[80,81]
eIF4g	1.0%	T436fs * 86; K643R	colon, lung (overexpression w/o genetic mutation)	[80,82,83]
mTOR	2.1%	S2215Y, S2215F, E1799K, T1977K, L1460P	colon, endometrium, skin, kidney	[80,84]
NF1	3.8%	R2450 *, R440 *, R1534 *	skin, soft tissue, urinary tract, lung, colon	[80,85,86]
PIK3CA	9.7%	H1047R, E545K, E542K, H1047L, Q546K, R88Q, N345K, C420L	breast, endometrium, urinary tract, colon	[80,87,88,89]
PIK3CG	1.6%	V759I, V165I, R472C, E267K, A84V	skin, colon, lung	[80,90,91]
PIK3R1	1.4%	N564D, R348 *, K567E, G376R	breast, endometrium, prostate, leukemia	[80,92]
PKC beta	1.0%	D427N, D630N, E533K	lung, skin, colon	[80,93]
PTEN	5.0%	R130G, R130Q, R233 *, R130 *	breast, endometrium, prostate, leukemia	[80,94,95]
Raptor	1.0%	R718C, R139H, Q1264fs * 4, T1121M	various	[80]
Rictor	1.0%	S1101L, R401C	lung, breast	[80,96]
TP53	25.2%	R175H, R248Q, R273H, R282W, R213 *, G245S, R249S, Y220C, R196 *, R342 *	solid cancer, leukemia, lymphoma, melanoma	[80,97,98]
TSC1	1.2%	M322T, P1143L	skin, urinary tract, liver	[80,99,100,101]
TSC2	1.7%	F690fs * 8, R1417fs * 59, S1364fs * 50, K1638 *	liver, breast	[80,101]
VHL	4.5%	kidney, neuroendocrine tumors	R161 *, L89H, S65 *	[80,102,103]

## Supplementary Materials

Supplementary materials can be found at http://www.mdpi.com/1422-0067/20/3/690/s1.
